# Time for a Measurement Check-Up: Testing the Couple’s Satisfaction Index and the Global Measure of Sexual Satisfaction Using Structural Equation Modeling and Item Response Theory

**DOI:** 10.1177/02654075221143360

**Published:** 2022-12-12

**Authors:** Christopher Quinn-Nilas

**Affiliations:** Department of Psychology, 7512 The Memorial University of Newfoundland, St. John’s, NL, Canada

**Keywords:** Structural equation modeling, item response theory, measurement, relationship satisfaction, sexual satisfaction

## Abstract

Relationship and sexual satisfaction are two central outcomes in the study of relationships and are commonly used in both academia and applied practice. However, relationship and sexual satisfaction measures infrequently undergo specific psychometric investigation. Ensuring that measures display strong psychometric performance is an important but under-tested element of replication that has come under more scrutiny lately, and adequate measurement of constructs is an important auxiliary assumption underpinning theory-testing empirical work. A measurement check-up was conducted, including Confirmatory Factor Analysis (CFA) to test factorial validity, measurement invariance to test for group comparability, and Item Response Theory (IRT) to assess the relationship between latent traits and their items/indicators. This format was used to evaluate the psychometric properties of the Couple’s Satisfaction Index (CSI) and the Global Measure of Sexual Satisfaction (GMSEX), two commonly used scales of relationship and sexual satisfaction with a sample of 640 midlife (40–59 years old) married Canadians who were recruited by Qualtrics Panels. Results of CFA suggested that both models were satisfactory. Invariance testing provided robust support for intercept invariance across all the groupings tested. IRT analysis supported the CSI and GMSEX, however, there was evidence that the GMSEX provided somewhat less information for those high on sexual satisfaction. This measurement check-up found that the CSI and GMSEX were reasonably healthy with some caveats. Implications are discussed in terms of replicability and meaning for scholars and practitioners.

## Introduction

Measurement issues have increasingly been in the spotlight as replicability and validity of findings are directly tied to the verity of one’s measurement instruments (e.g., Hussey & Hughes, 2020). Recent publications have documented that commonly used measures in psychology lack adequate validation efforts to support their ongoing use (Flake et al., 2017), highlighting a pervasive “measurement schmeasurement” attitude (Flake & Fried, 2020). Likewise, inattention to measurement invariance – an important assumption that scale scores are comparable and retain their meaning across groups (or timepoints) - has recently been labelled a “dire disregard” in psychological science (D’Urso et al., 2022). Increased attention to measurement has evolved out of the timely discussions of rigor and replicability in research. Increased attention to measurement has been long overdue - measurement issues have *always* been important because of substantial downstream effects on the interpretation of research results.

Relationship and sexual satisfaction are two central outcomes in the study of relationships and are commonly used in both academia and applied practice (McClelland, 2011; Wood et al., 2005) and their correlates are frequently the focus of research. However, in the fields of sexuality and relationships research, relationship and sexual satisfaction measures are infrequently the subject of specific psychometric investigation. If measures do not adequately and accurately capture the idea they are intended to measure, then inferences may ultimately be faulty regardless of how much effort and rigor is put into research design, statistical analysis, or sample size (Flake & Fried, 2020).

When measuring constructs via scales (often self-reported), researchers generate numerical representations that are assumed to take on a meaning that is in line with the intended conceptual idea – that scale scores reflect quantities of a construct. Testing this connective assumption via establishing and re-evaluating properties of the instruments themselves is a critical and ongoing process (Flake et al., 2017; Hussey & Hughes, 2020). Although considerable effort has gone into *establishing* initial psychometric evidence for scales in relationships and sexual science, far less has been done to *re-evaluate* it, specifically, factorial validity, measurement invariance, and more granular item assessment using Item Response Theory (IRT). Although studies like Mark et al. (2014) on sexual satisfaction and Funk and Rogge (2007) on relationship satisfaction specifically sought to evaluate these constructs and to ascertain which measures were better/best, researchers have been slow to build on these metric foundations with ongoing and more concentrated psychometric efforts. In short, measures in this field are infrequently given a measurement check-up - a periodic evaluation of the qualities of the measurement instruments to provide ongoing evidence supporting the factor structure, the group-comparability, and granular item performance of measures. I propose one possible configuration of this check-up: a combination of CFA to ascertain factorial validity, measurement invariance to evaluate evidence for/against group invariance across a wide selection of variables, and IRT to examine item and scale-based information indices (i.e., how much information is captured by the measure at different levels of the latent construct). I aimed to provide an assessment of the psychometric properties of two of the most used measurement instruments of relationship and sexual satisfaction - the Couple’s Satisfaction Index (CSI) and the Global Measure of Sexual Satisfaction (GMSEX) - in a sample of midlife married Canadians.

### Measurement Troubles in Relationships and Sexual Science

Recently it has been claimed that research in Psychology at large is characterized by a laisse faire attitude toward measurement – an attitude referred to as “measurement schmeasurement” (Flake & Fried, 2020, p. 1). This attitude can also be understood as a reliance on assumptions – such as the assumption that a rigorously developed measure maintains that “stamp” of rigor in perpetuity. Scale validation has never been a binary yes or no (Edwards et al., 2018), nor is it a permanent label that is earned once upon the measures’ creation and retained forever (Flake et al., 2017). This attitude exists in relationships and sexual science, too. Despite the importance of relationship and sexual satisfaction for the fields of close relationships and sexuality, investigations into the underlying metrics and specifically the factorial validity and invariance of relationship and sexual satisfaction are commonly overlooked. Researchers in relationship and sexual science predominantly use relationship and sexual satisfaction in studies to identify how they relate to other constructs, such as wellbeing (Holmberg et al., 2010) or mental health and stress (DeMontigny et al., 2020). Identifying associations and group differences with criterion variables of interest should be understood to be part of a larger framework of an ongoing and constant construct validation (Flake et al., 2017). The interconnection between relationship and sexual satisfaction has also been of considerable interest to researchers, as these topics share substantial conceptual and empirical overlap (cross-sectionally and longitudinally; McNulty et al., 2016; Quinn-Nilas, 2020). But it is critical to understand that the validity of the results of commonly used substantive analyses (e.g., correlation, regression, tests of mean differences) are predicated on the assumption that the measurement of the underlying constructs have adequate psychometric properties and that the numerical representations do indeed take on the intended meaning (Flake et al., 2017; Flake, 2021). Even if evidence continues to emerge that indicates our measures are of excellent quality, periodic check-ups of those measures should remain an important audit of foundational assumptions in our field.

### A Measurement Check-Up

#### Confirmatory Factor Analysis

Confirmatory Factor Analysis (CFA) is an element of the check-up that is focused on the structural phase of validity evidence (Flake et al., 2017). It assumes that the data generation mechanism is one in which the covariance of the items is a result of unobserved latent factor(s) which explain the covariance (Kline, 2015). Estimating CFA in structural equation modeling (SEM) software provides a gradient of support for the instruments’ factor structure which is an important part of overall validity of the measure. Likewise, measurement invariance is another part of the ‘structural’ phase of construct validity that underpins much comparative or substantive work (i.e., comparisons of relationship/sexual satisfaction across gender, precursor of tests of association like regression; Flake et al., 2017).

#### Measurement Invariance

To test measurement invariance is to test for evidence against the capacity of a scale to measure quantities of the same construct across different types of people (or across time) and in the same way (D’Urso et al., 2022; Vandenberg & Lance, 2000). If, for example, gender group comparisons are performed on variables like relationship and sexual satisfaction without first establishing measurement invariance across gender, the researcher (and the reader) cannot be certain whether the measure reflects the construct in meaningfully comparable ways across different gender groups (D’Urso et al., 2022; Flake et al., 2017). Without establishing measurement invariance across the groups of interest, it is extremely difficult to know what the observed numerical differences/similarities (i.e., those found from *t*-tests, ANOVA) are telling us about the phenomenon under consideration (D’Urso et al., 2022). The issues extend to experimental research. If relationship or sexual satisfaction are outcome variables of interest in these cases, invariance is critically important to establish between one’s control group and the experimental group to rule out possible threats to internal validity that may emerge due to assuming that the measure retains invariant properties post-intervention. Intervention could lead to changes in specific *scale items* rather than the intended changes to the *latent construct* (D’Urso et al., 2022)*.* In this case, comparing aggregated scores on that construct would obfuscate important nuances of the intervention. In the absence of evidence for measurement invariance scale scores can not solely be attributed to differences on the construct and may represent (to an unknown extent) group-based differences in measurement instrument functioning rather than quantities of the construct of interest.

Mean-level comparisons are by their nature founded on a measurement invariance assumption, but few studies explicitly test their measures for this property across the intended groups. In other words, researchers want to be making an ‘apples to apples’ comparison and not an ‘apples to sparkplugs’ comparison (Vandenberg & Lance, 2000). Alternatively, if evidence for noninvariance is presented, this might stimulate further inquiry into both the source of the noninvariance and perhaps prompt a reconceptualization of the construct and/or a re-evaluation of prior comparative findings (or, at minimum, an understanding that the measurement instrument is not understood similarly across groups).

Many different types of groups are compared within relationships and sexuality. For example, researchers have investigated gender differences in relationship and sexual satisfaction (Conradi et al., 2017; Mark & Murray, 2012), compared parents to non-parents (Schwenck et al., 2020), those who divorced and those who have not divorced (Diamond et al., 2018), and the effects of parental divorce (Amato & Booth, 1991; Feng et al., 1999). Additionally, variables such as age and marital duration are often utilized as covariates in studies examining relationship and sexual satisfaction - the utilization of such demographics for comparison or correlational purposes assumes invariance of the constructs (i.e., relationship and sexual satisfaction) across meaningful levels of these demographics. Each of these variables undergirds a substantial body of literature on its topic. In this study, the motivation to test invariance is founded dually on foundational and confirmatory rationales. In the foundational sense: researchers need to be equipped with empirical evidence supporting comparative work, regardless of if those variables/groups have or have not been tested (because they may be in the future). Additionally, evidence for noninvariance would present interesting avenues for future research into the sources of noninvariance. In a confirmatory sense: there are many studies that have investigated gender differences (Impett & Peplau, 2006), relationship duration differences (Abe & Oshio, 2018), parenthood (Twenge et al., 2003), age (Lee & McKinnish, 2018), parental divorce (Mustonen et al., 2011), and history of divorce (DeLongis & Zwicker, 2017) in sex and relationship satisfaction. Failure to establish measurement invariance between parents and non-parents on sexual satisfaction, as an example, would muddy the interpretation of any finding of statistically significant mean differences between these groups. Without measurement invariance, researchers cannot be sure whether observed differences reflect differing *quantities* of sexual satisfaction (i.e., the typical intention), or if they reflect differential functioning of the *instrument* between the groups (i.e., differing response styles; differing interpretations of scale items not attributable to sexual satisfaction). The absence of support for measurement invariance thus erodes the empirical strength of the inference that parents and non-parents differ on sexual satisfaction. This same conceptual example can be applied to each of the above variables of interest and underscores the importance of invariance. Regardless of whether studies have found evidence for differences or not in their statistical comparisons, the assumption of measurement invariance is part of the connective tissue between data analysis results (i.e., finding evidence/no evidence for differences) and substantive inference (i.e., attributing the finding of differences/no differences to differences/no differences in quantities of the construct) that should be made explicit.

#### Item Response Theory

Item response theory is the last piece of a check-up that will be discussed here. IRT is based on the idea that the difficulty and informational value of items on a test is a continuous variable that can be measured. By performing IRT, researchers can estimate the amount of information that each item and each scale on the test provides across the range of possible scores on the latent trait. This information can be used to improve the measurement precision of the test and to evaluate the quality of the items on the test. It has been increasingly used for the evaluation of psychological instruments and more recently for the development of some relationship and sexuality measures (e.g., Funk & Rogge, 2007; Shaw & Rogge, 2016). IRT provides additional empirical evidence toward the structural phase of construct validity (Flake et al., 2017) complimentary to the form of evidence provided by CFA, but additionally allowing a granular inference about the relative information provided by specific items. Thus, one facet of IRT relevant in this context is to identify if items and scales are informative (i.e., they capture suitable amounts of information about the latent construct) and to pinpoint specific items, specific response options, and specific scales that may be problematic (i.e., uninformative of the latent trait). With the importance of ongoing validation efforts and the anatomy of this measurement check-up established, I now turn to a review of psychometric evidence for the Couple’s Satisfaction Index (Funk & Rogge, 2007) and the Global Measure of Sexual Satisfaction (Lawrance & Byers, 1995).

### Relationship Satisfaction

Funk and Rogge (2007) provided convincing evidence that their Couple's Satisfaction Index (CSI) provides a greater depth of information about the relationship satisfaction construct than other comparable and popular measurement instruments (like the Dyad Adjustment Scale; Spanier, 1976) and the Marital Association Task (MAT; Locke & Wallace, 1959). The CSI measure became popular very quickly after it’s inception, amassing approximately 1, 379 citations on Google Scholar at the time of this study. Of these citations, using a search for “confirmatory factor analysis” suggests that almost all appear to be using CSI as a validity measure (i.e., not focused on checking the CSI itself) for the generation of new scales and/or the validation of *other* measurers. Only one study was found that specifically estimated CFA of the CSI: Forouzesh Yekta et al. (2017) conducted a CFA of a Persian translation of the CSI finding some support for its translated structure (i.e., some of the fit indices were “mixed”). No studies were found that assessed measurement invariance of this scale (and this is consistent with a systematic review of relationship quality measurement performed by Ribotta et al., 2021). Additionally, no instances of the CSI being subjected to IRT beyond what was originally performed by Funk and Rogge (2007) as part of the development of the scale were found. Importantly, the sample used by Funk and Rogge (2007) was of relatively young participants (average age was 27 years old). The scale’s psychometric performance among older married populations has not yet been separately tested.

### Sexual Satisfaction

A host of measures purport to examine sexual satisfaction. One measure, the GMSEX which was developed as part of a larger model called the Interpersonal Exchange Model of Sexual Satisfaction (Lawrance & Byers, 1995), appears to have garnered popularity as it is highly cited (N = 778) and the measure has been republished in several editions of the Handbook of Sexuality-Related Measures (e.g., Milhausen et al., 2019). Additionally, recent psychometric-focused studies have recommended its use (i.e., Mark et al., 2014). A similar search for “confirmatory factor analysis” of the citing studies, and also using the Handbook of Sexuality Related Measures (Milhausen et al., 2019) as a source, produced few studies. Sánchez-Fuentes et al. (2015) validated a Spanish version of the IEMSSQ which consists of a two-factor model that includes the GMSEX questions alongside GMREL questions (a measure of relationship satisfaction; Lawrance & Byers, 1995) and Calvillo et al. (2020) similarly provided support for the IEMSSQ model with a Spanish sample of gay men and lesbian women. Both studies found some support for the GMSEX measure (with some fit indices that were acceptable rather than excellent by conventional standards). Calvillo et al. (2020) did find evidence for measurement invariance across sexual orientation and biological sex. These two validation studies -- the only validation studies of the GMSEX found -- were focused on validating translations of the scales. Translations should indeed be validated, but the original scale itself should also get a check-up to ensure that the content being translated is worth the translation. This underscores a broader point: current validation efforts of the original version are needed, too. Additionally, few studies assessed measurement invariance of this scale making the basis for group-comparisons on this scale unclear. Although Mark et al. (2014) concluded by recommending the GMSEX if a unidimensional measure of sexual satisfaction was desired, additional validation efforts using CFA and including invariance testing will buttress these conclusions (Mark et al., 2014, did not focus on structural validity). Thus, the current study is a continuation and extension of this line of work. The only instance of the GMSEX being subjected to IRT was in Shaw and Rogge (2016) - which presented reasonably optimistic findings although, as noted, their sample was comprised of relatively young individuals leaving the performance of this scale among older married adults unclear.

### Rationale

In brief, it appears that ongoing validation of the CSI and GMSEX has been minimal. A recent wave of literature (D’Urso et al., 2022; Flake & Fried, 2020; Flake et al., 2017; Hussey & Hughes, 2020) has motivated a more skeptical and analytic approach to measurement: assume less; test more. The small number of metric studies on the CSI and GMSEX means their structural validity (i.e., factorial structure and invariance) is uncertain and underscores the broader point– researchers often make assumptions about the verity of the instruments being used and cite a small handful of studies (and Cronbach’s alpha) in support of their measures of choice (Flake et al., 2017; Flake & Fried, 2020).

As such, in the current study, I conducted the following tests to lay bare evidence for these assumptions with a sample of midlife (age 40–59) married Canadians:

(1) CFA measurement models of the CSI and the GMSEX.
(2) Measurement invariance testing across a wide range of potential sources of noninvariance including (a) age, (b) gender, (c) parenthood, (d) parental divorce, (e) relationship length, and d) participant history of divorce/separation.
(3) IRT focusing on the analysis of Test Information Curves to ascertain how much (and what types) of information about satisfaction are captured by the CSI and GMSEX scales (and their underlying items).

## Method

### Procedure

Following Research Ethics Board approval, participants were recruited through Qualtrics analytics panels. Qualified participants – individuals who were between the ages of 40–59, residing in Canada, and who were married (no restrictions based on age at marriage or length of marriage) – were sent a basic invitation to the survey. Interested participants clicked a link and proceeded to the survey package. The survey was accessed by approximately 1917 people – the survey was made inaccessible once data from 700 participants who met the study criteria was acquired. We retained a high standard for the data – any participant who incorrectly answered any of the four attention check questions was discarded (*n*_removed_ = 6). Also, analysis of unusual responses was conducted specifically to look for uniform response and cases were removed if the analysis showed that the participant responded in such a way (*n*_removed_ = 3). Lastly, cases that utilized “choose not to respond” for too many items (5% or more) were also discarded (*n*_removed_ = 31), alongside responses that were too quick (i.e., faster than half the median completion speed; *n*_removed_ = 20). The final sample included 640 individuals.

### Measures

#### Demographic Variables

Participants answered demographic questions assessing their age, gender, sexual orientation, education, province of residence, parenthood (and number of children), and ethnicity. Given that several demographic variables were the focus of invariance testing, Table 1 contains information detailing how these variables were scored, how they were grouped, and the group sizes.

**Table 1.** Summary of Invariance Testing Variables.

Grouping Variables	Original Response Options	Grouping Method	Details	Group Sizes
Relationship length	Numeric	Median split	Above and below median (Med = 19)	Below median: 311 Above median: 329
Parenting	Yes/No	Dichotomization	Yes children/No children	Parent: 514 Not parent: 124
Age	Numeric	Median split	Above and below median (Med = 45)	Below median: 302 Above median: 338
Parental divorce	Yes/No	Dichotomization	Parents divorced/Parents not divorced	Parents divorced: 131 Not divorced: 503
Gender	Woman (cisgender and transgender), man (cisgender and transgender), gender queer, non-binary, agender	Dichotomization	Men/Women (inclusive of cisgender and transgender)	Man: 272 Woman: 324
History of divorce/Separation	Separated, divorced, widowed, none	Dichotomization	Yes divorced or separated ever/not divorced or separated ever	Yes: 144 No: 483

*Note*. For gender, there were 25 individuals who indicated gender queer, non-binary, or agender and 16 individuals who chose not to respond. These individuals were retained for the CFA and IRT models, but due to it being an insufficiently large group to test for invariance they were excluded from the gender invariance testing (but retained in other invariance tests).

#### Sexual Satisfaction

The Global Measure of Sexual Satisfaction (GMSEX; Lawrence & Byers, 1995) was used to assess sexual satisfaction. The scale asks, “In general, how would you describe your relationship with your partner?” and the participant is presented with five dimensions, each along a seven-point scale: Good – Bad, Pleasant – Unpleasant, Positive – Negative, Satisfying – Unsatisfying, and Valuable – Worthless. Higher scores on this scale indicate higher sexual satisfaction. Internal consistency has consistently been high (α > .90; Byers, 2005; Lawrance & Byers, 1995). Internal consistency in the present study was excellent (see Table 2)

**Table 2.** Descriptive Statistics of CSI and GMSEX Mean Scores and Individual Items.

	*M (SD*)	Min-Max	Skewness	Kurtosis	Cronbach’s Alpha	Omega	AVE	Factor Loading	*R* ^*2*^
CSI	4.44 (1.35)	1–6.25	−.624	−.482	.954	.956	.843		
Item 1	4.54 (1.42)	1–7	−.56	−.22				.82	.67
Item 2	4.44 (1.48)	1–6	−.59	−.74				.92	.84
Item 3	4.35 (1.42)	1–6	−.57	−.57				.96	.92
Item 4	4.41 (1.44)	1–6	−.65	−.52				.97	.94
GMSEX	5.10 (1.65)	1–7	−.760	−.179	.966	.966	.850		
Item 1	4.92 (1.81)	1–7	−.68	−.43				.86	.78
Item 2	5.25 (1.65)	1–7	−.92	.23				.93	.86
Item 3	5.11 (1.72)	1–7	−.79	−.20				.97	.93
Item 4	4.97 (1.85)	1–7	−.76	−.43				.92	.84
Item 5	5.25 (1.77)	1–7	−.99	.14				.92	.85

*Note*. AVE = Average variance extracted.

#### Relationship Satisfaction

Relationship satisfaction was assessed using the Couple’s Satisfaction Index – four Item version (CSI; Funk & Rogge, 2007). The measure was developed using 180 items from some of the most commonly used relationship and marital satisfaction scales; these items were pooled and analyzed using principal components analysis and Item Response Theory. The CSI is a single dimensional measure with three versions: a 36-item, a 16-item, and a four-item version, and their IRT analysis supports that the 36-item and 16-item contain more information and measure relationship satisfaction better than all of the other commonly used measures (the four-item version being equal in quality even to the lengthiest of the other tested scales). An example item from the CSI is “In general, how often do you think that things between you and your partner are going well?” The measure has excellent internal consistency (α = .98; Funk & Rogge, 2007), strong convergent validity with existing relationship satisfaction scales (r = .89 with the 32 item DAS; Funk & Rogge, 2007), and strong construct validity with other scales such as ineffective arguing and neuroticism that are mirrored by other well-known relationship satisfaction scales (Funk & Rogge, 2007). The four-item version was used here and had excellent internal consistency (see Table 2).

### Data Analysis

The *lavaan* package for R was used to estimate the various CFA models. This sample exceeds sample size cutoffs established by simulation studies (i.e., Wolf et al., 2013). Each model was estimated using a robust estimator (MLR in *lavaan*) and with missing data handled using Full Information Maximum Likelihood (FIML; see Enders, 2010). No residual covariances were specified in any models. No modification indices nor any post-hoc modifications were done to improve fit - the analysis was approached from a confirmatory perspective. Evaluation of model fit was performed from two perspectives. The first was based on more traditional fit cut-off criteria and followed Hussey and Hughes (2020), to be denoted “mixed”, indices must be as follows: SRMR ≤.09 and any of: CFI ≥.95, TLI ≥.95, or RMSEA ≤.06. The second and primary perspective was based on dynamic fit cut-offs as described by McNeish and Wolf (2021), which uses simulation methods to estimate model fit cut-offs that are tailored for the specifics of the model. A specific issue that this ameliorates is that traditional fit cut-offs, like those for RMSEA, SRMR, and CFI are inaccurate particularly in the single factor context (the context at hand in this study). For transparency, I report model fit inferences based on both the traditional cut-off criteria and dynamic cut-off criteria (estimated using the application developed by Wolf & McNeish, 2020) but focus inferences on the dynamic fit cut-offs. Because the goals of this study were to ascertain metric qualities, additional indices such as factor loadings and average variance extracted (AVE) were also examined.

Measurement invariance was appraised using multi-group CFA and three levels of invariance were assessed: configural invariance, loading invariance, and intercept invariance which each reflect an increasingly strict criteria for measurement invariance. Typically intercept invariance is considered the baseline for reasonably comparable groups. Model fit criteria for measurement invariance were ΔCFI > −.15 and ΔRMSEA <.01 (see Chen, 2007) – invariance was supported if model fit did not deteriorate an amount greater than this criterion step-to-step.

Polytomous item response theory (via the Graded Response Model) was estimated using the *ltm* package in R (Rizopoulos, 2006). Similarly to other studies in this field (e.g., Funk & Rogge, 2007; Shaw & Rogge, 2016) inferences were focused on interpretation of the Item Information Curves (IICs; i.e., a visualization of information captured by each item along the spectrum of the latent trait under consideration) and Test Information Curves (TICs; i.e., a summative visualization of the information from the IICs to a single visual that shows the amount of information captured by the whole scale across the spectrum of values of the latent trait) which were estimated from a constrained discrimination polytomous model. IRT is a large sample technique (minimum of 500 subjects; Clark & Watson, 1995; this sample exceeds this minimum) that evaluates scales by estimating a latent score for each participant on the construct. In the case of this study, this would mean that each participant has a latent score estimated for both relationship satisfaction and sexual satisfaction. Latent trait estimates for IRT curves are displayed at + or – four SD above/below the mean. All output and code for SEM and IRT models can be found on the author’s OSF page (https://osf.io/tbhws/?view_only=211c8f2137b34cd8a8ed5f3a402b006d).

The Omega coefficient – a more optimal index of internal consistency reliability (compared to Cronbach’s alpha) was estimated in *lavaan* (see Hayes & Coutts, 2020 for details). The Omega coefficient is interpreted similarly to Cronbach’s alpha in that a cut-off of above .70 is recommended.

## Results

### Sample Description

Most participants were from Ontario (*n* = 286; 44.7%), Alberta (*n* = 82; 12.8%) and British Columbia (*n* = 74; 11.6), whereas Prince Edward Island (*n* = 4; .6%) and the Northwest Territories (*n* = 1; .2%) were the least represented. Participants were largely “White” (*n* = 530; 83%), with the second and third largest groups being “Southeast Asian” (*n* = 42; 6.6%) and “Black” (*n* = 16; 2.5%). The sample was mostly heterosexual (*n* = 593; 92.8%) with a smaller number of people were bisexual (*n* = 23; 3.6%). For gender, the sample was almost equally distributed between women (including cisgender and transgender; *n* = 324; 54%) and men (cisgender and transgender; *n* = 275; 46%). Twenty-five individuals indicated that they were gender queer, agender, or gender non-binary. In terms of sexual orientation, the sample was largely heterosexual (*n* = 592; 93%). Overall, the sample was predominantly college/university educated (*n* = 275; 43%), but substantial minority proportions had reported that their highest education level was high school (*n* = 104; 16.3%) and trade/technical/vocational training (*n* = 52; 8.1%). The average age was 49.80 (*SD* = 5.89) years, and most participants had children (*n* = 514; 81%). Of those with children, the average number of children was 2.12, with 27% (*n* = 137) of parents reporting having one child, 45% (*n* = 229) of parents reporting having two children, and 19% (*n* = 99) having three children with a smaller proportion having more than three children (*n* = 44; 8.6%). All participants were currently married, and the average duration of these marriages was 18.34 years (*SD* = 9.75).

### Measurement models

Both the CSI and the GMSEX measurement models had mixed support according to traditional fit cut-offs (as shown in Table 3). Based strictly on traditional fit indices, models were evaluated as having mixed fit because RMSEA and the *X*^2^ were below acceptable by conventional standards (i.e., *X*^2^ was significant and RMSEA was not below .06 nor was the 90% ULCI under .08). Based on dynamic fit indices, both measurement models had good fit. Factor loadings and *R*^2^ for all items were excellent. Average Variance Extracted (AVE) values - the averaged standardized factor loadings - were strong for both scales exceeding the commonly held cut-off of .50. In summary, although inferences based on traditional fit cut-offs alone suggested mixed empirical support (using the Hussey & Hughes, 2020 classification heuristic) of the CSI and GMSEX, inferences based on dynamic fit indices, factor loadings, *R*^2^ and AVE suggest strength in the factor models.

**Table 3.** Fit Indices for Measurement Models.

	*X*^2^ (df), *p*-value	CFI	TLI	RMSEA 90%CI [LLCI, ULCI]	SRMR	BIC	Traditional Model Fit	Dynamic Model Fit
Relationship satisfaction	14.76 (2), *p* = .001	.996	.987	.101 [ .057, .152]	.007	6204.669	Mixed	Good
Sexual satisfaction	22.258 (5), *p* < .001	.986	.973	.132 [ .079, .19]	.013	8759.525	Mixed	Good (see note)

*Note*. For traditional: Good confirmatory model fit meets all of: CFI ≥.95, TLI ≥.95, RMSEA ≤.06, and SRMR ≤.09. To be denoted “mixed”, indices must be as follows: SRMR ≤.09 and any of: CFI ≥.95, TLI ≥.95, or RMSEA ≤.06.

For dynamic relationship satisfaction cut-offs: CFI ≥.991, TLI ≥.95, RMSEA ≤.147, and SRMR ≤.012.

For dynamic sexual satisfaction cut-offs, the conservative cut-offs (corresponding to a 95% of misspecified models will be correctly rejected, and a 5% of correctly specified models incorrectly rejected when 1/3 of the items have an un-modeled residual correlation of .3) would be: Good confirmatory model fit meets all of: CFI ≥.995, TLI ≥.95, RMSEA ≤.079, and SRMR ≤ .009. The more liberal dynamic cut-offs (corresponding to a 95% of misspecified models will be correctly rejected, and a 5% of correctly specified models incorrectly rejected when 2/3 of the items have an un-modeled residual correlation of .3): Good confirmatory model fit meets all of: CFI ≥.985, TLI ≥.95, RMSEA ≤.134, and SRMR ≤.013. This latter cut-off threshold was used.

### Measurement Invariance

Specifics regarding how data were categorized are shown in Table 1. Fit indices for each invariance model can be seen in Tables 4 and 5, and summary inferences can be seen in Table 6. Every model met the criteria required for comparisons between groups (i.e., intercept invariance). However, as noted in Tables 4 and 5, model fit of almost all groups was mixed based on traditional fit cut-offs. Dynamic fit indices provided broad support for the model fit of almost all models (only one was mixed using these cut-offs). Together, results suggest that there was empirical evidence to support comparability across groups.

**Table 4.** Results of Measurement Invariance for Relationship Satisfaction (CSI-4).

	*X*^2^ (df), *p*-value	CFI	RMSEA 90%CI [LLCI, ULCI]	BIC	Δ *X*^2^	ΔCFI	ΔRMSEA	Traditional Model Fit	Dynamic Fit
Rel length									
Config	15.099 (4), *p* = .004	.996	.095 [.047, .148]	6260.126				Mixed	Good
Loadings	18.166 (7), *p* = 0.011	.996	.069 [.031, .109]	6242.515	3.067	.000	−.026	Mixed	Good
Intercepts	23.368 (10), *p* = .009	.996	.064 [.030, .098]	6228.322	5.202	.000	−.005	Mixed	Good
Child									
Config	18.925 (4), *p* = .001	.995	.103 [.060, .152]	6253.673				Mixed	Good
Loadings	21.542 (7), *p* = .003	.996	.077 [.041, .115]	6236.473	2.617	.001	−.026	Mixed	Good
Intercepts	29.137 (7), *p* = .001	.994	.073 [.043, .104]	6223.457	7.595	−.002	−.004	Mixed	Good
Age									
Config	15.674 (4), *p* = .003	.996	.096 [.049, .148]	6275.152				Mixed	Good
Loadings	17.521 (7), *p* = .014	.997	.067 [.028, .106]	6256.505	1.847	.001	−.029	Mixed	Good
Intercepts	17.618 (10), *p* = .062	.998	.048 [.000, .084]	6237.481	.097	.001	−.019	Good	Good
Parental divorce									
Config	23.941 (4), *p* < .001	.994	.120 [.076, .168]	6196.836				Mixed	Good
Loadings	31.151 (7), *p* < .001	.993	.098 [.064, .134]	6182.989	7.21	−.001	−.022	Mixed	Good
Intercepts	29.987 (10), *p* = .001	.994	.076 [.046, .108]	6163.649	1.164	.001	−.022	Mixed	Good
Gender									
Config	18.516 (4), *p* = .001	.995	.111 [.063, .165]	5826.219				Mixed	Good
Loadings	19.990 (7), *p* = .006	.996	.077 [.039, .118]	5807.285	1.474	.001	−.034	Mixed	Good
Intercepts	31.665 (10), *p* < .001	.992	.084 [.052, .118]	5800.000	11.675	−.004	.007	Mixed	Good
History of sep/Div									
Config	17.239 (4), *p* = .002	.996	.101 [.055, .153]	6105.924				Mixed	Good
Loadings	21.221 (7), *p* = .003	.996	.076 [.040, .114]	6088.839	3.982	.000	−.025	Mixed	Good
Intercepts	28.077 (10), *p* = .002	.995	.071 [.041, .103]	6075.289	6.856	−.001	−.005	Mixed	Good

*Note*. Good confirmatory model fit meets all of: CFI ≥.95, RMSEA ≤.06. Mixed means CFI ≥.95 or RMSEA ≤.06 but not both. The same dynamic fit cut-offs shown in Table 2 are used to evaluate the dynamic model fit.

**Table 5.** Results of measurement invariance for Sexual Satisfaction (GMSEX).

	*X*^2^ (df), *p*-value	CFI	RMSEA 90%CI [LLCI, ULCI]	BIC	Δ *X*^2^	ΔCFI	ΔRMSEA	Factor Structure	Dynamic Fit
Rel length									
Config	27.556 (10), *p* = .002	.987	.130 [.073, .189]	8822.092				Mixed	Good
Loadings	36.675 (14), *p* = 0.001	.986	.113 [.069, .159]	8804.670	9.119	−.001	−.017	Mixed	Good
Intercepts	44.077 (18), *p* = .001	.986	.100 [.063, .137]	8782.683	7.402	.000	−.013	Mixed	Good
Child									
Config	27.335 (10), *p* = .002	.988	.123 [.069, .180]	8796.072				Mixed	Good
Loadings	32.563 (14), *p* = .003	.989	.100 [.055, .146]	8772.447	5.228	.001	−.023	Mixed	Good
Intercepts	40.351 (18), *p* = .001	.988	.090 [.053, .128]	8752.091	7.788	−.001	−.01	Mixed	Good
Age									
Config	28.343 (10), *p* = .002	.987	.131 [.075, .188]	8828.154				Mixed	Good
Loadings	36.952 (14), *p* = .014	.986	.113 [.069, .158]	8809.645	8.609	−.001	−.018	Mixed	Good
Intercepts	47.425 (18), *p* < .001	.984	.105 [0.069, .141]	8793.790	10.473	−.002	−.008	Mixed	Good
Parental divorce									
Config	23.941 (10), *p* < .001	.994	.138 [.085, .195]	8763.625				Mixed	Mixed
Loadings	38.083 (14), *p* = .001	.985	.115 [.072, .160]	8741.015	14.142	.009	−.023	Mixed	Good
Intercepts	44.112 (18), *p* = .001	.986	.099 [.062, .136]	8716.304	6.029	.001	−.016	Mixed	Good
Gender									
Config	20.788 (10), *p* = .023	.991	.106 [.038, .171]	8283.777				Mixed	Good
Loadings	31.313 (14), *p* = .005	.988	.103 [.054, .152]	8273.336	10.525	−.003	−.003	Mixed	Good
Intercepts	41.406 (18), *p* < .001	.986	.098 [.059, .137]	8258.504	10.093	−.002	−.005	Mixed	Good
History of sep/Div									
Config	25.141 (10), *p* = .005	.988	.124 [.064, .185]	8675.481				Mixed	Good
Loadings	35.210 (14), *p* = .001	.986	.111 [.066, .157]	8659.598	10.069	−.002	−.013	Mixed	Good
Intercepts	41.223 (18), *p* = .002	.987	.095 [.057, .134]	8635.288	6.013	.001	−.016	Mixed	Good

*Note*. Good confirmatory model fit meets all of: CFI ≥.95, RMSEA ≤.06. Mixed means CFI ≥.95 or RMSEA ≤.06. The same dynamic fit cut-offs shown in Table 2 are used to evaluate the dynamic model fit using the 95%/5% error split under the assumption that 2/3 of the items have residual correlations that were not modeled.

**Table 6.** Summary of Invariance Testing Results for CSI.

Variable	Measurement invariance	Test Failed
Couple’s Satisfaction Index (CSI; 4-item)		
Rel length	Passed	NA
Child	Passed	NA
Age	Passed	NA
Parental divorce	Passed	NA
Gender	Passed	NA
History of divorce/Separation	Passed	NA
Global Measure of Sexual Satisfaction (GMSEX)		
Rel length	Passed	NA
Child	Passed	NA
Age	Passed	NA
Parental divorce	Passed	NA
Gender	Passed	NA
History of divorce/Separation	Passed	NA

*Note*. Passing requires meeting configural invariance, loading invariance, and intercept invariance (threshold for failure: ΔCFI > −.15 and ΔRMSEA <.01; see Chen, 2007).

### Overall Summary of Measurement Models and Invariance

In summary, findings suggest good empirical evidence supporting the CSI and GMSEX for this midlife married sample according to dynamic fit indices, and strong evidence according to factor loadings, *R*^2^, and AVE. Based on traditional cut-offs the measurement models were mixed. Invariance testing supported that both scales were intercept invariant across all the grouping variables: relationship length, children, age, parental divorce, gender, history of divorce/separation. These results present empirical evidence in favor of measurement invariance across these groups and supporting their compatibility.

### Item Response Theory

**Figure 1.**
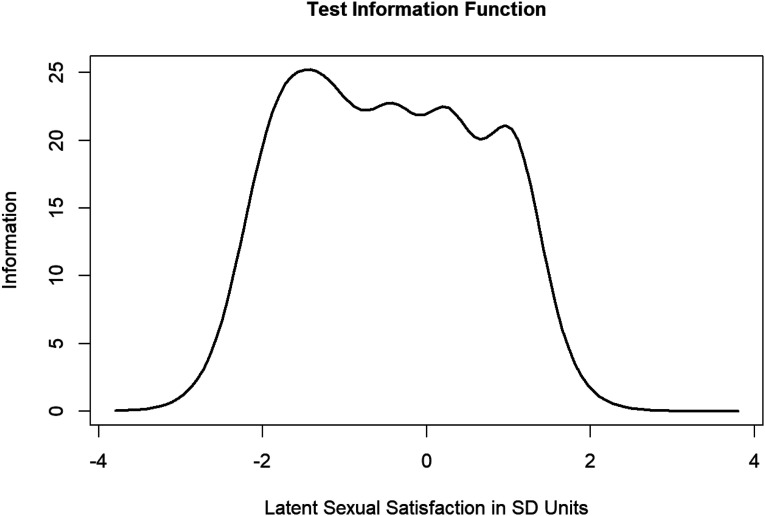
Test Information Curve (TIC) for sexual satisfaction measured by the GMSEX.

**Figure 2.**
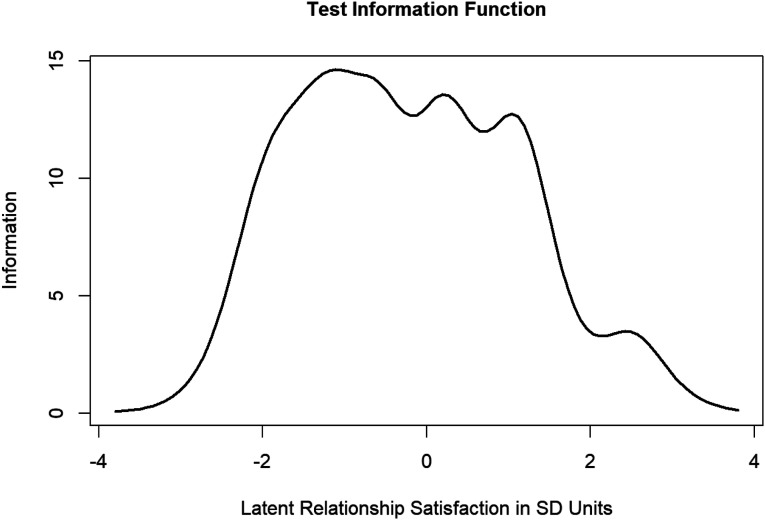
Test Information Curve (TIC) for relationship satisfaction measured by the CSI.

The primary aim was to examine if the CSI and GMSEX items and scales were effective at capturing the full spectrum of scores across the response categories (i.e., low satisfaction to high satisfaction). To investigate this, Test Information Function Curves (TICs; Figures 1 and 2) were plotted and the amount of information captured by the tests under specific ranges (between −4 and 0 SD was quantified as “lower” trait, and between 0 and four SD was quantified as “higher” trait) was computed. When visually inspecting TICs, ideally the lines should peak around the mean (0 on the *x*-axis). TICs suggested that the GMSEX (Figure 1) provided more information about sexual satisfaction for lower levels of the latent construct compared to higher levels (the scale captures approximately 63% of the total information of low latent trait levels vs. 37% of total information for high trait levels). Visually, the line does indeed peak at around the mean with a bias toward peaking at the lower ends of the scale. Therefore, IRT suggested that the GMSEX was better at capturing information on low levels of sexual satisfaction compared to higher levels but generally performed well. Scale performance was more balanced for the CSI (Figure 2), with it being roughly similarly effective at capturing information at high and low levels of relationship satisfaction (the scale captures approximately 57% of the total information of low latent trait levels vs. 43% of total information for high trait levels). Visual inspection supports that the information does peak around the mean with a bias toward peaking at the lower ends of the scale. IRT suggested that the CSI was reasonably balanced at capturing information from high versus low levels of relationship satisfaction, though it was somewhat better at capturing low relationship satisfaction information. This slight bias toward capturing more information for lower satisfaction participants is also observable when inspecting the Item Information Curves (IICs; see OSF page). However, in all, inspection of the various information curves paints an optimistic picture of the performance of the CSI and the GMSEX scales (and the individual items) with the caveat being that both appear better at capturing information for those lower on satisfaction (to varying degrees between them).

## Discussion

Effective measures require regular check-ups -- even the most established measures should be evaluated periodically to maintain a strong evidence base supporting their use. Decisions about the quality of measurement instruments should not be a “yes or no” binary - quality of measurement is a matter of degrees (Edwards et al., 2018) based on an available evidence base that should evolve and grow over time. Re-evaluating evidence supporting scales via a measurement check-up as proposed here requires an embrace of inferential complexity (i.e., beyond a yes or no dichotomy) and a humble acknowledgement that some measures may not perform as well as hoped. Researchers who study relationship and sexual satisfaction rarely test the instruments themselves, meaning that even scales that are widely used and considered ‘high quality’ have largely unknown metric properties. My goal was to provide one data point in a trajectory of longer-term effort to assess commonly used relationship and sexuality scales. Findings provide support for both the CSI and GMSEX in midlife married populations, strong support for metric invariance across all the tested groups (age, gender, parenthood, parental divorce, relationship length, and participant history of divorce/separation), and good metric properties as found with IRT analyses. Caveats include potential issues with the GMSEX providing somewhat less information on low satisfaction individuals.

As stated by Sakaluk (2019), the types of theory testing performed in substantive analyses rely on a set of auxiliary assumptions about the world, including assumptions about measurement. Additionally, invariance testing can and should be seen as an interesting and material outcome of interest for theory (Sakaluk, 2019). It should not simply be a hurdle that one must begrudgingly vault over before testing the “real theory” but rather, part of a larger marathon of research into the nuances of measurement. Highly granular analyses of items and scales, as are available using techniques shown here should be regularly conducted to buttress the results of both metric and substantive work.

Relationship and sexual satisfaction are constructs that are frequently used in clinical settings; thus, utilizing measures that strike an appropriate balance between length, empirical support for construct validity, and can detect adequate amounts of information across the full range of levels of the satisfaction trait of interest is of great importance. For example, one would not want to evaluate satisfaction levels using a scale, only to find that the scale is insensitive to individuals on the very low end of the satisfaction spectrum. Additionally, one would not want to utilize a scale to inform treatment, only to find that it does not measure what it purports to measure because then it is unclear what the score actually “means.” For researchers, having measures that are valid and informative is important for statistical power, and for longitudinal studies of change which necessitate the use of measures that are adequately sensitive to change (and across the full spectrum of scaling). In short, it has never been sufficient to evaluate scales on face-validity alone, nor on Cronbach’s alpha, and systematic psychometric check-ups like this one have a broadly supportive effect for scholars and practitioners.

### CFA Findings

By traditional fit indices the measurement models were of mixed fit, but by dynamic fit indices they were of good fit. As Lai and Green (2016) indicated, it is quite common to encounter scenarios in which many different fit indices disagree based on traditional cut-offs – placing researchers in a puzzling situation. Is there something wrong with the measures of fit, or is something wrong with the data? Worse still, is something wrong with the standards used to evaluate fit? Lai and Green (2016, p. 232) succinctly states this about fit index conflicts: “*When two watches disagree to a nontrivial extent, one is confused about the time. After reasons for the disagreement are figured out, it does not necessarily follow that one will know the time – it could be that both watches are dysfunctional.*” The idea of abandoning fixed goodness-of-fit cut-offs has been debated considerably in the methodological literature because a one-size-fits-all approach is difficult to justify with models as complex as those studied in structural equation modeling (Groskurth et al., 2021). Dynamic fit indices are proposed as a method to tailor the cut-offs to the specific structure of the tested model using a simulation methodology (McNeish & Wolf, 2021). Although this method is new it presents many appealing conceptual and methodological features above and beyond the traditional cut-offs. Given that the method is still new, I present inferences based on both the traditional and dynamic assessments in the results of this paper but weigh inferences primarily upon the dynamic cut-offs. The goal in this study was not to weigh in on a heavy debate on the efficacy of fit indices, rather, I take a broader perspective that acknowledges that our goal as relationships and sexual scientists is not necessarily to find a perfectly fitting model, but to acknowledge the aphorism that all models are wrong, but some are useful (Box, 1976).

### Measurement Invariance Findings

Results from tests of measurement invariance provide broad support for invariance across six different grouping variables: relationship length, children, age, parental divorce, gender, and history of divorce/separation. These findings are supportive of prior and ongoing substantive and comparative work using the CSI and GMSEX scales. Regardless of relationship duration, whether the individual is a parent, age group, whether their parents divorced, their gender, and their own history of divorce or separation – evidence presented in this study does not suggest that CSI and GMSEX are interpreted differently across these groups. Thus, empirical evidence is presented to support that mean-level differences observed can be taken to indicate differences on the construct and not idiosyncratic differences on the instrument. Of course, studies seeking to test such differences are cautioned to test invariance with their samples. Analyzing these variables in relation to relationship satisfaction (as measured by the CSI) and sexual satisfaction (as measured by the GMSEX) are predicated on invariance assumptions (and assumptions that the measurement models function as good representations of their constructs). As this is just one study with one data set, researchers should continue to examine the validity of these commonly used measures and continue to replicate findings of invariance and to expand the testing of invariance beyond just the groupings shown here.

### Item Response Theory Findings

Findings from IRT analyses were largely supportive of the CSI and the GMSEX. It is not surprising that the CSI, which was developed using IRT (Funk & Rogge, 2007) would perform well here; well-performing, highly informative items were deliberately chosen in constructing the scale (Funk & Rogge, 2007). Regarding the GMSEX, Shaw and Rogge (2016) was the only study to our knowledge that used IRT on the GMSEX. They found that the GMSEX was reasonably effective at measuring across all levels of the trait (particularly for its length), however, in the current study GMSEX captured more information at low levels of satisfaction than it did for high levels of satisfaction. This divergence may be due to important sample differences – notably, that mean age of the current sample was much higher and all participants were married (compared to 29% married in Shaw & Rogge, 2016). These are important sampling differences, and divergent findings may suggest that that the items exhibit differential functioning based on age, relationship type, and/or relationship/marital duration (when considered in a broader context of young adults vs. midlife adults). Alternatively, although the sample size in this study does meet heuristic cut-offs minimums for statistical power with IRT, Shaw and Rogge (2016) had a much larger sample. IRT results from a larger sample of midlife married adults may ultimately converge on the results of Shaw and Rogge (2016). It is also possible that because most participants scored very highly on the GMSEX that there was limited variability to be detected on the higher end of the scoring spectrum. Regardless, these possibilities should be explored in future studies seeking to replicate these findings.

## Future Directions

Relationships and sexuality researchers are often concerned with unobservable (i.e., latent) constructs (e.g., satisfaction) under the assumption that the “latent variable exerts influence at the level of the individual” (Borsboom et al., 2003, p. 203). Much is assumed of our measures - it is important to regularly give our measures a check-up to evaluate if our assumptions hold up to increased scrutiny. Incorporating the estimation of measurement models alongside the tests of substantive hypotheses – what is done when one utilizes latent variable models within a broader SEM framework - will allow for more transparency of measurement and more publicly visible evidence for (or against) measures.

This is not the only form a measurement check-up could take - methods in this area are constantly evolving and new methods regularly emerge (for example, the dynamic fit indices). The form demonstrated in this study, involving CFA, measurement invariance testing, and IRT is one that utilizes methods that are widely used and cover a wide breadth of measurement topics of interest for applied researchers (i.e., factorial validity, group-comparability, item performance). Importantly, developments in the field of psychometric network modeling are predicated on a different data-generation model: that the indicators have direct relations between one another, and factors emerge because of local clustering between items (instead of being due to a latent variable’s causal forces). Future research should approach the topic of psychometrics in relationships and sexuality science from a network perspective (Isvoranu et al., 2022) as there are likely many constructs that could be reasoned to be generated by such a mechanism.

### Limitations

This study is hopefully one of a larger stream of studies tasked with validating measures in this field. Results shown here may be idiosyncratic to the sample (or to statistical noise) and so replication is needed. These findings, on their own, should not be taken as a strict endorsement/nonendorsement of these specific measures. Some of the comparisons for invariance testing, like the parenting, history of divorce, and parental history of divorce are based on very unequal sample sizes and thus these findings should be replicated in future research with better distributions (see Table 2). Additionally, IRT is a particularly sample-size intensive analysis. Some of the inferences here may be limited due to sample size. In addition, the data in this sample is skewed toward those reasonably and highly satisfied with their relationship and their sexual relations. This is both a sampling issue - a larger sample is needed to garner enough low satisfaction participants, but also a selection bias issue. Specifically, by looking at midlife marriages, participants may inadvertently be a sample from a group that skews away from low satisfaction marriages (because some proportion of low satisfaction marriage may separate or divorce by this time). One variable that is clearly missing from the invariance testing results is sexual orientation and/or relationship configuration. Unfortunately, our sample was almost entirely heterosexual leaving the sample of individuals with more diverse sexual orientations severely lacking sufficient sample size to test invariance across these groups. This is a critical next step for research because researchers should not assume that scales of satisfaction - which are often developed and validated initially with predominantly heterosexual samples - carry the same meaning for individuals in more diverse relationship configurations. It should be noted that this study consisted solely of Canadians. It is possible that there may be non-invariance of relationship and sexual satisfaction across countries of residence, and this should be tested in future studies as well. There is of course a risk that some participants had their marital partners also participate as separate cases (which would violate independence). Given this is an issue with most (if not all) anonymous online studies on relationships there is no reason why this is a larger or more probable issue in this study.

## Conclusion

Analysis of the Couple’s Satisfaction Index (CSI) and Global Measure of Sexual Satisfaction (GMSEX) in a sample of midlife Canadians found support for their psychometric properties. An important caveat was that the GMSEX (and to a lesser extent the CSI) appeared to capture more information on low satisfaction participants than high. Intercept invariance was demonstrated across several variables relevant for researchers in this field. Broadly, this study suggests that the CSI and the GMSEX scales perform well in a sample of midlife, married, primarily heterosexual individuals, however future work should examine further these instruments (and other instruments) in this (and other) populations. Researchers and practitioners seeking short but psychometrically sound scales to evaluate relationship or sexual satisfaction in this population should consider the CSI and the GMSEX but with cognizance of their limitations (and the limitations of this study).
